# Identification of novel metabolism-related biomarkers of Kawasaki disease by integrating single-cell RNA sequencing analysis and machine learning algorithms

**DOI:** 10.3389/fimmu.2025.1541939

**Published:** 2025-04-10

**Authors:** Chenhui Feng, Zhimiao Wei, Xiaohui Li

**Affiliations:** ^1^ Capital Institute of Pediatrics-Peking University Teaching Hospital, Beijing, China; ^2^ Department of Cardiovascular Medicine, Children’s Hospital Capital Institute of Pediatrics, Beijing, China

**Keywords:** Kawasaki disease, bile acid metabolism, fatty acid metabolism, single-cell RNA sequencing, machine learning

## Abstract

**Background:**

The bile acid metabolism (BAM) and fatty acid metabolism (FAM) have been implicated in Kawasaki disease (KD), but their precise mechanisms remain unclear. Identifying signature cells and genes related to BAM and FAM could offer a deeper understanding of their role in the pathogenesis of KD.

**Method:**

We analyzed the public single-cell RNA sequencing (scRNA-seq) dataset GSE1687323 to characterize the immune cell-type landscape in KD. Gene sets related to BAM and FAM were collected from the Gene Set Enrichment Analysis (GSEA) database and previous literature. We analyzed the cellular heterogeneity of BAM and FAM at the single-cell level using R packages. Through differential expressed genes (DEG) analysis, high-dimensional Weighted Correlation Network Analysis (hdWGCNA) and machine learning algorithms, we identified signature genes associated with both BAM and FAM. The cellular expression patterns of signature genes were further validated using our own scRNA-seq dataset. Finally, quantitative real-time PCR (qRT–PCR) was performed to validate the expression levels of signature genes in KD, and Receiver Operating Characteristic (ROC) curve analysis was conducted to evaluate their diagnostic potential.

**Results:**

Enhanced BAM and FAM were detected in monocytes and natural killer (NK) cells from KD in the public scRNA-seq dataset. Our scRNA-seq data confirmed the signature genes identified by machine learning algorithms: Vimentin (VIM) and chloride intracellular channel 1 (CLIC1) were upregulated in monocytes, while integrin subunit beta 2 (ITGB2) was elevated in NK cells of KD. qRT-PCR results also validated the bioinformatic analysis. Moreover, these genes demonstrated significant diagnostic potential. In the training dataset (GSE68004), the area under the curve (AUC) values and 95% CI were as follows: VIM: 0.914 (0.863–0.966), ITGB2: 0.958 (0.925–0.991), and CLIC1: 0.985 (0.969–1). The validation dataset (GSE73461) yielded similarly robust results, with AUC values and 95% CI: VIM: 0.872 (0.811–0.934), ITGB2: 0.861 (0.795–0.928), and CLIC1: 0.893 (0.837–0.948).

**Conclusion:**

This study successfully identified and validated VIM and CLIC1 in monocytes, as well as ITGB2 in NK cells, as novel metabolism-related genes in KD. These findings suggest that BAM and FAM may play crucial roles in KD pathogenesis. Furthermore, these signature genes hold promising potential as diagnostic biomarkers for KD.

## 1 Introduction

Kawasaki disease (KD), also known as mucocutaneous lymph node syndrome, predominantly affects children under 5 years old. The main symptoms include fever, polymorphic rash, conjunctival congestion, cervical lymphadenopathy, oral mucosa and extremities lesions, etc. (1). However, many patients do not exhibit classic clinical features, leading to delays in diagnostic and treatment and increasing the risk of coronary artery complications. Therefore, elucidating the pathogenesis of KD and identifying potential molecular biomarkers are crucial for the timely and accurate diagnosis, effective treatment, and prevention of adverse outcomes.

Bile acid metabolism (BAM) and fatty acid metabolism (FAM) have been implicated in the development and progression of various diseases (2, 3). Shao et al. (4) reported a strong association between the upregulation of BAM and FAM and increased expression levels of cytokines and inflammation-related genes in the macrophages of COVID-19 patients. Clinical studies have demonstrated a significant correlation between fasting serum total bile acid (BA) levels and the severity of coronary heart disease, where activation of bile acid receptors promotes cholesterol clearance and exerts anti-atherosclerosis effects (5). During the acute phase of KD, elevated serum total BA levels may serve as a potential biomarker for predicting IVIG treatment responsiveness (6). Zhu Q et al. (7) reported that palmitic acid, a fatty acid compound, could exacerbate endothelial cell apoptosis by fostering the production of reactive oxygen species in KD. Our previous study also revealed an upregulation of bile acid and fatty acid in KD (unpublished). Collectively, these studies suggest a potential role of BAM and FAM in KD. Previous study have indicated that α-linolenic and linoleic acid (LA) are related to elevated isolithocholic acid levels, suggesting a possible crosstalk between BAM and FAM (8). However, the precise pathological mechanisms of BAM and FAM in KD, as well as the potential crosstalk between them, remain unclear.

Advances in sequencing technologies have facilitated the widespread application of single-cell RNA sequencing (scRNA-seq) in the biomedical research, establishing it as a crucial tool for analyzing cellular heterogeneity and deepening our understanding of disease pathogenesis (9). The integration of machine learning algorithms with other bioinformatics approaches offers the prospect of identifying disease biomarker based on pathogenesis (10, 11). In this study, we adopted a novel approach by integrating scRNA-seq and bulk RNA-seq data to identify cellular subpopulations and core genes with BAM and FAM upregulation in KD. Using machine learning algorithms, we further refined these identified genes to reveal signature genes that may serve as candidate metabolic biomarkers for the early diagnosis of KD.

## 2 Materials and methods

### 2.1 Data source

The scRNA-seq dataset, including 9 samples from 3 healthy controls (HC) and 6 KD patients, was obtained from the GEO database (Accession ID: GSE1687323) (12). The six KD patients were between 1.6 and 5.4 years old, with an equal male-to-female ratio (1:1). None of the KD patients had coronary artery lesions (CALs) and one of them was diagnosed with incomplete KD.

Two bulk RNA-seq datasets GSE68004 (13) and GSE73461 (14) containing samples from both HC and KD children were also collected from the GEO database. The GSE68004 dataset included 44 male and 32 female KD patients, aged between 3 and 138.6 months. All of them were complete KD and 12 patients companied with CALs. In the GSE73461 dataset, there are 43 male and 35 females KD patients, with a median age of 27 (16–45) months. All of them were complete KD and 33 patients companied with CALs. Among these datasets, GSE68004 was used as the training set while GSE73461 was employed for validation. Detailed information of all datasets used in this study is provided in **Supplementary Table 1**. Additionally, to validate the results from public database analysis, we utilized our previous single-cell data (15). Finally, we incorporated 6 complete KD patients (4 with CALs (KL) and 2 without CALs) and 4 healthy controls, with a male-to-female ratio of 1:1 and aged 0.6-4.6 years old.

### 2.2 Gene set source

BAM-related genes were retrieved from the Gene Set Enrichment Analysis (GSEA) database (http://www.gsea-msigdb.org/gsea/index.jsp) using the search term “bile acid”, resulting in the identification of four BAM-related gene sets (KEGG, REACTOME, GO, Hallmark MSigDB v5.2). Moreover, a set of BAM-related genes was obtained from a previous study (16). In total, 189 BAM-related genes were included for analysis in this study (**Supplementary Table 2**). In addition, 92 FMA-related genes was gathered for further analysis based on previous research (**Supplementary Table 3**) (17).

### 2.3 Data processing

The raw data were processed into a Seurat object via the Seurat R package (18, 19). The following conditions are indicative of low-quality cells (1): fewer than 200 or more than 7000 detected genes (2), mitochondrial gene expression exceeding 20% of the total, or (3) red blood cell gene expression below 3%. Following normalization, 52,681 high-quality cells were retained for further exploration. The data was then normalized using the Harmony package (20) to eliminate batch effects (21). The “FindVariableFeatures” function was applied to identify the top 3000 most variable genes. Principal component analysis (PCA) was subsequently performed to reduce the dimensionality of the scRNA-seq data, using the top 3000 most variable genes as input. The “FindCluster” function was then used for clustering, with the resolution parameter set to 0.8 (22). The clustering results were visualized using UMAP. To identify marker genes for various cell clusters, the “FindAllMarkers” function from Seurat was applied to distinguish cells within specific clusters from all other cells. Known biological cell types were annotated based on typical marker genes (**Supplementary Table 4**). The internal queue was analyzed using the same approach. For bulk RNA-seq data, samples who did not meet the criteria for complete KD were removed.

### 2.4 Signaling pathway score

BAM and FAM scores at the single-cell level for all samples in GSE1687323 were evaluated using the AUCell (23), UCell (24), Singscore (25), ssGSEA (26), and AddModuleScore (27) algorithms. To refine BAM or FAM scores, we applied Z-score standardization and Min-Max normalization to the raw score matrix, first transforming the features to a zero-mean, unit variance scale, and then rescaling them to the range [0,1] for consistency. The combined BAM or FAM scores were derived by summing the standardized feature values row-wise (28). All cells from HC and KD were then classified into three groups: those with a high combined BAM or FAM score exceeding 75%, those with a low combined BAM or FAM score below 25%, and the remaining cells classified as the median BAM or FAM group based on the quartile value of the total score. Correlation analysis was performed between high BAM and FAM cells. Subsequently, based on the previously computed BAM and FAM scores, cells were further classified into three groups: those both exhibiting high BAM and FAM scores, those with both low BAM and FAM scores and the remaining cells designated as the median group. The “FindMarkers “ function was employed to conduct a differential expressed genes (DEGs) analysis, identifying differential genes between high BAM/FAM and low BAM/FAM cells (29).

### 2.5 High-dimensional weighted correlation network analysis (hdWGCNA)

A co-expression network was constructed in high BAM and FAM cells of both healthy controls and KD patients based on single-cell level data using the “hdWGCNA” package (30). The hdWGCNA enables the construction of cell-type specific co-expression networks, the identification of robust gene modules, and the establishment of a biological framework for these modules. The genes expression profiles related to high BAM and FAM modules were explored by eigengene-based connectivity (kME). The “ConstructNetwork” function was utilized to generate the co-expression network. All analyses followed the official standard procedure, as detailed in https://smorabit.github.io/hdWGCNA/articles/hdWGCNA.html.

### 2.6 Protein–protein interaction analysis and enrichment analysis

The STRING database (https://string-db.org/) was used to assess protein-protein interaction (PPI) interactions among the hub genes identified through DEG and hdWGCNA analyses. The “organism” parameter was set to “Homo sapiens,” while all other parameters were left at their default settings. In the results, nodes represent genes and edges denote interactions between them. Pathway and functional enrichment analyses of these genes were conducted using Gene Ontology (GO) and Disease Ontology (DO).

### 2.7 Machine learning algorithms identify the signature genes

We employed seven machine learning algorithms (LASSO, SVM-RFE, Boruta, RandomForest (RF), Decision tree (DTs), XGBoost and Gradient Boosting Machine (GBM)) to identify the signature genes associated with BAM and FAM upregulation.

LASSO regression was implemented for feature selection, with the optimal regularization parameter identified through 10-fold cross-validation. The glmnet package was used to train the model using binary logistic regression (family=binomial) and L1 regularization (alpha=1). A regularization path comprising 100λ values was generated, and the λ value that minimized cross-validation error (lambda.min) was chosen for the final model. To ensure reproducibility in cross-validation data partitioning, a random seed of 123 was set. The model automatically standardized features and retained only those with non-zero coefficients. Feature importance was evaluated via 10-fold cross-validation, with SVM-RFE iteratively removing the 10 least important features per iteration (k=10). In each subsequent iteration, the number of removed features was halved. A linear kernel SVM was used to compute feature weights, with importance determined by the average ranking (AvgRank) across 10 cross-validation runs. An error rate curve was generated for all feature subsets, and the subset with the lowest error rate (optimal count: 22) was selected. Feature importance was assessed using RF algorithm with 500 decision trees (ntree = 500).At each node split, √p features were randomly selected (where p is the total feature count). Model performance was evaluated by out-of-bag (OOB) error, with the optimal tree count determined by minimizing OOB error. Features were ranked based on their mean decrease in Gini impurity (MeanDecreaseGini), with the top 50 genes retained. The RF package was used to generate error curves and gene importance rankings. XGBoost was applied with a tree depth of 15, a learning rate of 0.2, and 50 iterations. Feature importance was ranked based on gain metrics. Boruta was executed with 1,000 iterations to resolve uncertain features, leveraging shadow features for comparative decision plots. GBM was trained using 4-fold repeated cross-validation with a default tree depth of 1 and 100 iterations. Log2-transformed feature importance scores were reported. A minimally pruned classification tree was constructed using a complexity parameter of 1e-10 in DTs to maintain its full structure. Feature importance was determined based on node purity improvement. By integrative the results of these machine learning algorithms by a Venn plot, we identified the optimal signature genes associated with BAM and FAM upregulation.

### 2.8 Gene set enrichment analysis of the signature genes

In the bulk RNA-seq dataset, we conducted gene set enrichment analysis (GSEA) on the signature genes to evaluate their relevance to specific biological processes or disease states.

### 2.9 Revaluated the signature genes at the cellular level

To validate our findings, we analyze signature genes in our own scRNA-seq data. Through the analysis of annotated scRNA-seq data, we examined the distribution of these signature genes across different cell subtypes. This in-depth analysis identified the specific cell types in which these genes play a crucial role in regulating both BAM and FAM.

### 2.10 Trajectory analysis

Cell trajectory inference was performed using the Monocle 2 algorithm (31). Following dimension reduction and single-cell sequencing, differentiation trajectories were inferred using standard parameters. We also performed Cellular Trajectory Reconstruction Analysis (CytoTRACE) using default parameters (21, 32), leveraging the well-established principle that transcriptional diversity decreases during differentiation. This algorithm predicts differentiation states on scRNA-seq data, complementing the trajectory analysis conducted with Monocle2.

### 2.11 Interactions between intercellular communication and transcription factors

The CellChat (33) approach was employed to assess variations in intercellular communication modules by inferring, analyzing, and visualizing hypothesized receptor-ligand signaling interactions. Cell type-specific interactions were identified by detecting over-expressed ligands or receptors within the cell group, followed by the recognition of enhanced ligand-receptor interactions.

### 2.12 RNA extraction and qRT-PCR analysis

The study was approved by the Ethics Committee. From November 2023 to December 2024, three KD patients and three healthy controls were recruited from the Children’s Hospital Capital Institute of Pediatrics. Peripheral blood mononuclear cells (PBMCs) were isolated from patient blood samples as described previously (15). Total RNA was extracted from PBMCs using the Tissue Total RNA Isolation Kit (TIANGEN, Beijing). The extracted RNA was reverse transcribed into cDNA using the PrimeScript™ RT Reagent Kit and gDNA Eraser. Quantitative real-time PCR (qRT-PCR) was conducted using real-time PCR instruments, with GAPDH as the endogenous control for mRNA. The reaction conditions were as follows: pre-denaturation at 95°C for 30 seconds, denaturation at 95°C for 10 seconds, and annealing at 60°C for 30 seconds (40 cycles). The amplification of target genes and the internal reference gene was performed separately for each sample. Each sample was analyzed in triplicate. Data analysis was performed using the 2^(-ΔΔCt) method. Differences between the HC and KD groups were assessed using a two-sided Student’s *T*-test. The primer sequences used in this study are provided in **Supplementary Table 13**.

### 2.13 The expression and predictive significance of the signature genes in KD

The Wilcoxon rank-sum test was used to analyze the expression of signature genes in the training set samples (GSE68004). To assess the diagnosis value of these signature genes, the area under the receiver operating characteristic (ROC) curve was calculated (34). The same approach was then applied to verify the results in the validation dataset (GSE73461).

### 2.14 Statistical analysis

All data processing and statistical analysis were conducted using R version 4.1.3 (package versions detailed in **Supplementary Table 14**) and GraphPad Prism 10.4 statistical software (USA). The data was presented as the standard error of the mean ± mean (SEM). Differences in continuous variables between two groups were assessed using either the Wilcoxon test or t-test, while comparisons among multiple groups were evaluated by one-way analysis of variance test (ANOVA). Correlation between variables was analyzed using Pearson’s correlation coefficient. All *p*-values were calculated using the two-tailed tests, with statistical significance defined as *p* < 0.05.

## 3 Results

### 3.1 BAM and FAM are upregulated in monocytes and NK cells in KD

**Figure 1:**
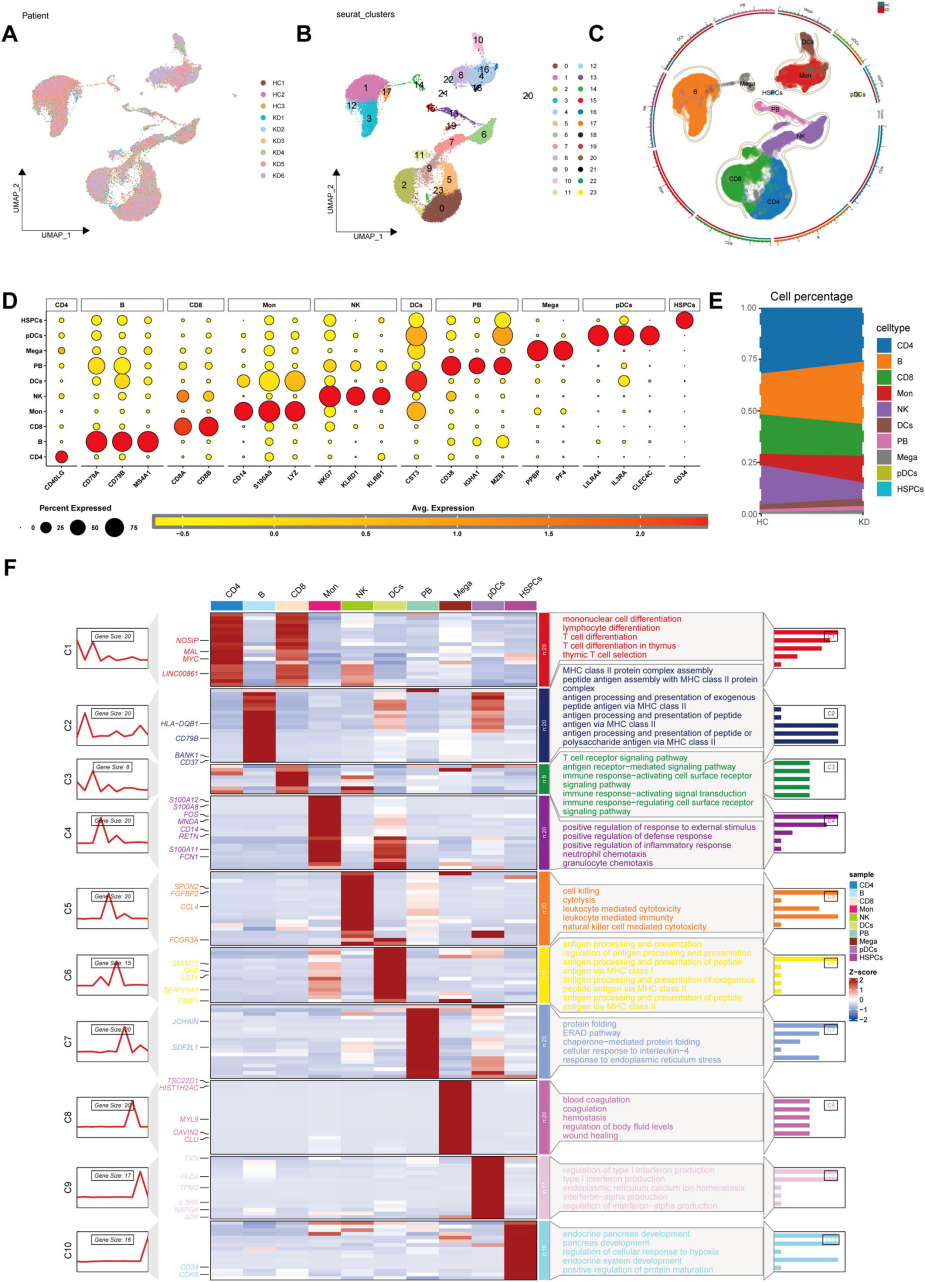
Screening of public single-cell data. **(A)** Principal component analysis (PCA) revealed a consistent cellular distribution across all samples. **(B)** Uniform manifold approximation and projection (UMAP) plot delineated 23 distinct cellular clusters precisely. **(C)** The UMAP of cells from the public scRNA-seq dataset, colored by cell-type annotation. **(D)** Dot plot showing typical marker genes for each cell type. **(E)** The proportion of each cell type between KD and HC. **(F)** The heat map showing the relationship between marker genes across the 10 identified cell types, complemented by Gene Ontology (GO) pathway enrichment analysis. HC, healthy control; KD, Kawasaki disease; DC, dendritic cell; Mono, monocyte; Mega, megakaryocyte; NK, natural killer; pDC, plasmacytoid dendritic cells; HSPC, hematopoietic stem and progenitor cells.

Following quality control, we identified 52,681 high-quality cells from GSE1687323 that were eligible for further analysis. Upon reanalyzing the single-cell data, we identified 10 distinct cell lineages based on marker genes: CD4^+^ T cells (CD40LG), B cells (MS4A1, CD79A, CD79B), CD8^+^ T cells (CD8A, CD8B), monocytes (CD14, S100A9, LYZ), natural killer (NK) cells (KLRB1, NKG7, KLRD1), dendritic cells (DCs) (CST3), plasmablasts (CD38, IGHA1, MZB1), megakaryocytes (PPBP, PF4), plasmacytoid dendritic cells (pDCs) (LILRA4, IL3RA, CLEC4C) and hematopoietic stem and progenitor cells (HSPCs) (CD34) (**Figures 1A–D**, **Supplementary Figures 1A–C**), consistent with Wang’s study (12). Notably, a high proportion of monocytes and B cells was observed in KD (**Figure 1E**). GO analysis provided a comprehensive insight into the characteristics of these cell types based on their respective marker genes (**Figure 1F**).

**Figure 2:**
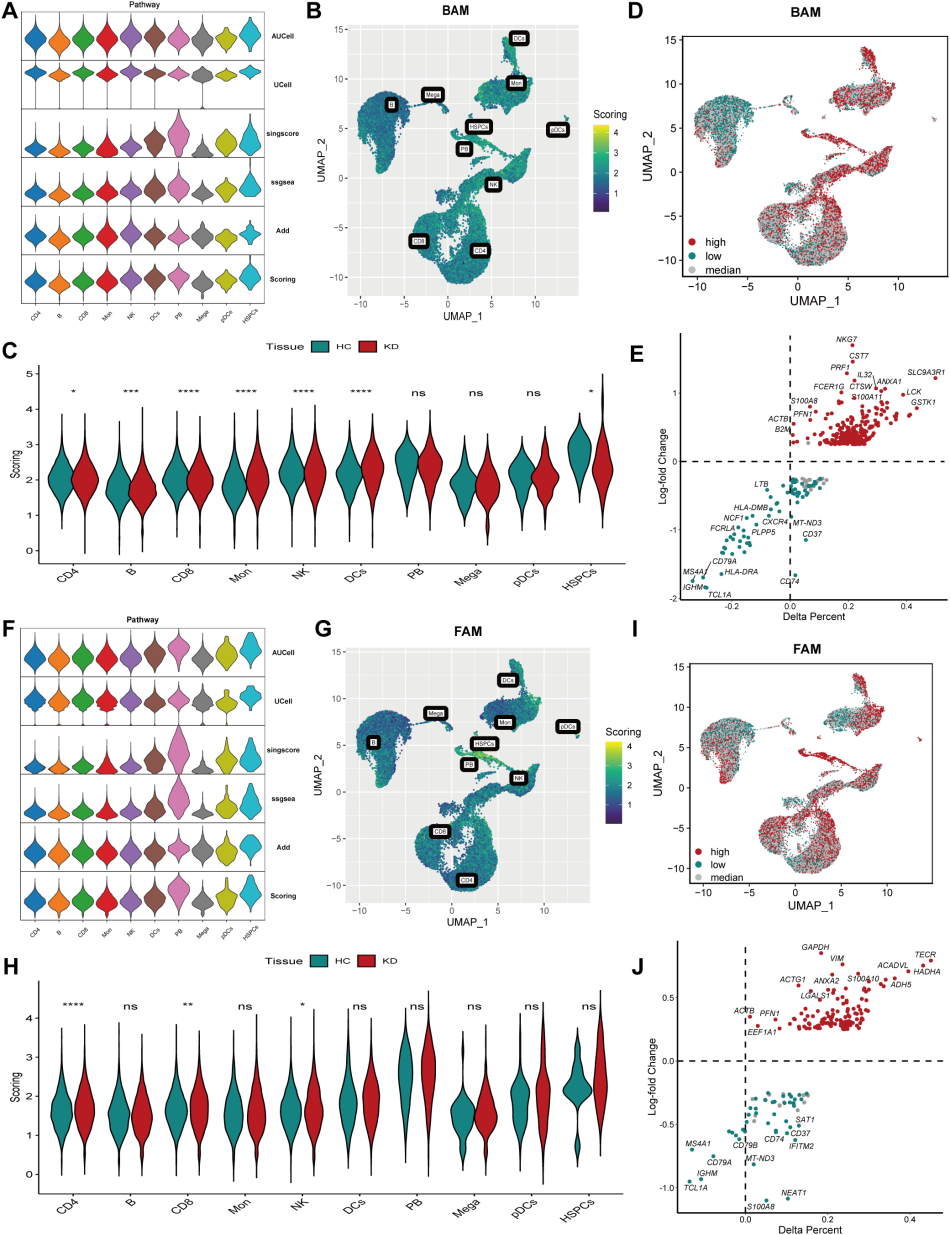
Analysis of bile acid metabolism (BAM) and fatty acid metabolism (FAM) in Kawasaki disease (KD). **(A)** The violin plot showed BAM score in different cell subtypes calculated by AUCell, UCell, singscore, ssGSEA and AddModuleScore algorithms. **(B)** The UMAP visualized BAM score in different cell subtypes. **(C)** The violin plot demonstrated BAM score in different cell subtypes in KD and healthy controls. Significant differences were determined with Wilcoxon test (*p*>0.05 (ns), *p ≤ 0.05, **p ≤ 0.01, ***p ≤ 0.001, ****p ≤ 0.0001). **(D)** The UMAP plot illustrating three groups: high BAM score cells, low BAM score cells and median cell groups based on quartile of the BAM score. **(E)** The result of differential expression gene **(DEG)** analysis associated with BAM. **(F)** The violin plot showed FAM score in different cell subtypes. **(G)** The UMAP visualized FAM score in different cell subtypes. **(H)** The violin plot demonstrated FAM score in different cell subtypes in KD and healthy controls. Significant differences were determined with Wilcoxon test (*p*>0.05 (ns), *p ≤ 0.05, **p ≤ 0.01, ***p ≤ 0.001, ****p ≤ 0.0001). **(I)**: The UMAP plot illustrating three groups: high FAM score cells, low FAM score cells and median cell groups based on quartile of the FAM score. **(J)**: The volcano plot showing differential expression gene (DEG) results associated with FAM. HC, healthy control; KD, Kawasaki disease; BAM, bile acid metabolism; FAM, fatty acid metabolism; UMAP, Uniform manifold approximation and projection. *p*>0.05 (ns), *p ≤ 0.05, **p ≤ 0.01, ***p ≤ 0.001, ****p ≤ 0.0001.

To examine BAM at single-cell level, we computed BAM score for each cell using multiple algorithms. The results revealed heterogeneous BAM profiles among different cell populations in both healthy controls and KD patients. Specifically, monocytes, DC cells and NK cells exhibited elevated BAM scores, while B cells, plasmablasts and CD8^+^ T cell displayed decreased BAM scores in KD (**Figures 2A–C**). Based on the BAM scores, all cells were classified into three groups according to quartile values: 13170 high BAM cells (scores exceeding 75 percentage), 13170 low BAM cells (scores below 25 percentage), and 26341 median BAM cells (**Figure 2D**, **Supplementary Figure 2A**). DEGs analysis between the high BAM score cells and low BAM score cells identified 257 genes associated with BAM upregulation (**Figure 2E**, **Supplementary Table 5**).

Subsequently, FAM was analyzed using the same methodology. The results revealed increased FAM scores in monocytes, DC cells, plasmablasts and NK cells, whereas B cells and CD8^+^ T cells exhibited decreased FAM scores (**Figures 2F–H**). Similarly, cells were also divided into three groups: 13170 high FAM cells, 13170 low FAM cells, and 26341 median FAM cells (**Figure 2I**, **Supplementary Figure 2B**). Additionally, DEGs analysis identified 120 genes associated with FAM upregulation (**Figure 2J**, **Supplementary Table 6**).

Based on the preceding outcomes, we conducted correlation analysis between high BAM and FAM cells, revealing a strong correlation (**Supplementary Figures 2C, D** **).** The cells were then categorized into three groups: 5137 cells with both high BAM and FAM scores named as High_BAM_FAM cells, 4999 cells with both low BAM and FAM scores named as Low_BAM_FAM cells, and the remaining cells classified as median cell groups (**Supplementary Figure 2E**). The metabolic plasticity of these cells may reflect the specific characteristics of different cell types.

### 3.2 HdWGCNA identified genes upregulating both BAM and FAM

**Figure 3:**
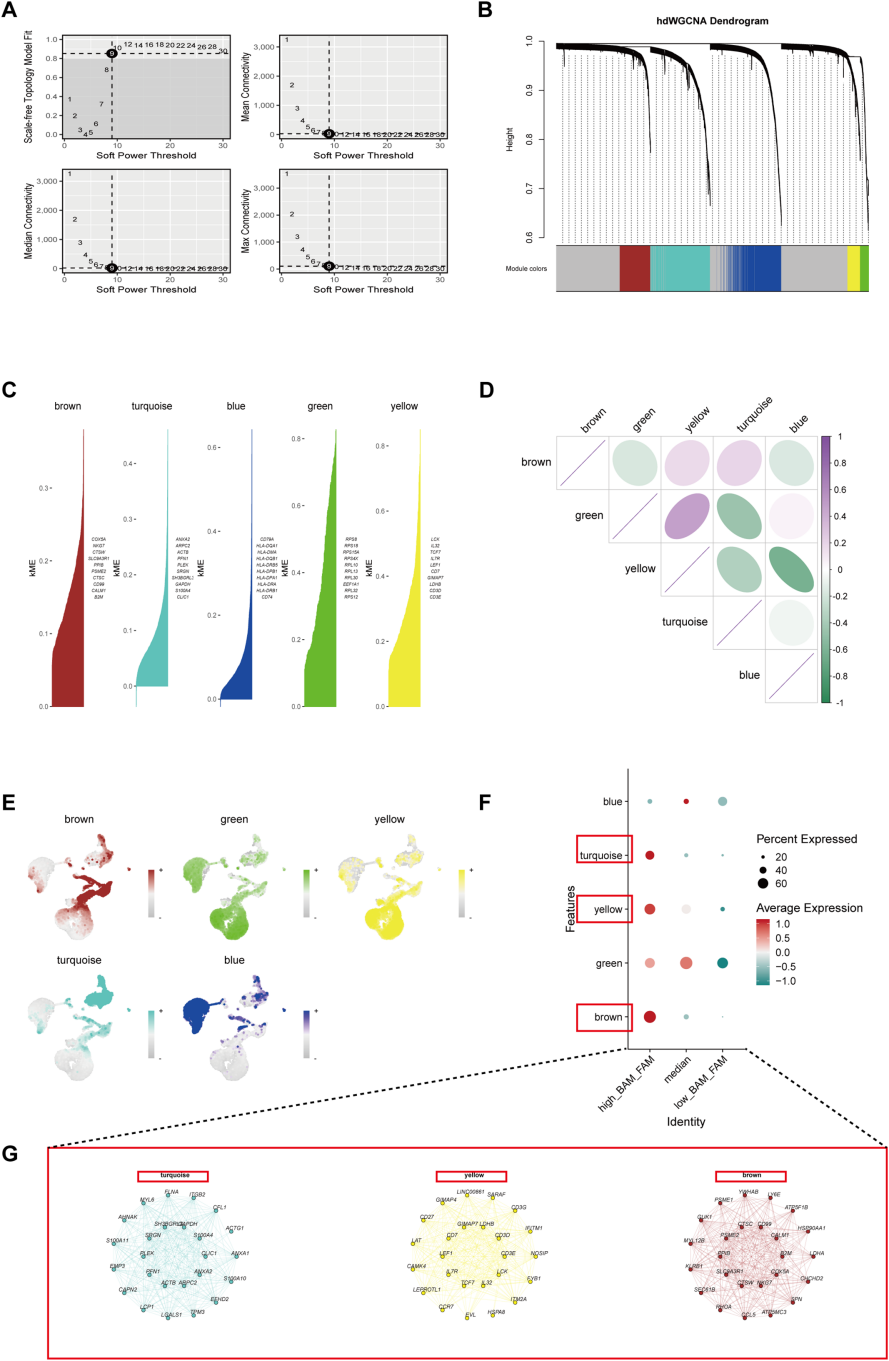
Identification of the crucial modules related to BAM and FAM by hdWGCNA. **(A)** The top left panel indicated the choice of the soft power threshold to achieve a fit of the scale-free topology model ≥0.9. The other three panels showing the mean, median and maximum connectivity of the topological network against various minimum soft thresholds. **(B)** The hdWGCNA dendrogram showing five modules identified. **(C)** Five modules were obtained and the top genes in each module were verified and ranked by kME (eigengene-based connectivity). **(D)** The relationship analysis between different modules. **(E)** The UMAP plots showing the expression density of the five modules. **(F)** A bubble plot represented the scores of the five modules in different metabolic cell groups. **(G)** Networks of the representative genes from turquoise, yellow and brown module.

To gain deeper insights into key markers in high BAM and FAM cells, we conducted hdWGCNA analysis with a soft power value of 9 to construct the co-expression network, identifying five distinct gene co-expression modules (**Figures 3A, B**). The top 10 most influential genes within these modules were determined based on kME values (**Figure 3C**). Correlation analysis of the modules revealed strong positive associations among the turquoise, yellow, and brown modules (**Figure 3D**). Moreover, the distribution of these modules across different cells types demonstrated high expression of the turquoise, yellow, and brown modules in high_BAM_FAM cells (**Figures 3E, F**). To investigate the functional roles of genes within these three modules, we selected a total of 408 genes with kME above 0.2, which were more likely to play central regulatory roles within their respective modules (**Supplementary Table 7**), with hub genes presented in **Figure 3G**. Notably, within these modules, CD3E, LEF1, and ACTB have been previously reported to be associated with BAM (35, 36). CTSW, LDHB, and IL32 have been reported to be linked to FAM (37–39). Additionally, SLC9A3R1 and GAPDH are implicated in the regulation of both BAM and FAM (40–43). These findings not only supported the robustness of our results but also suggested the potential role of BAM and FAM in the pathogenesis of KD.

### 3.3 Identifying a hub gene set named Up_BAM_FAM_hdWGCNA

**Figure 4:**
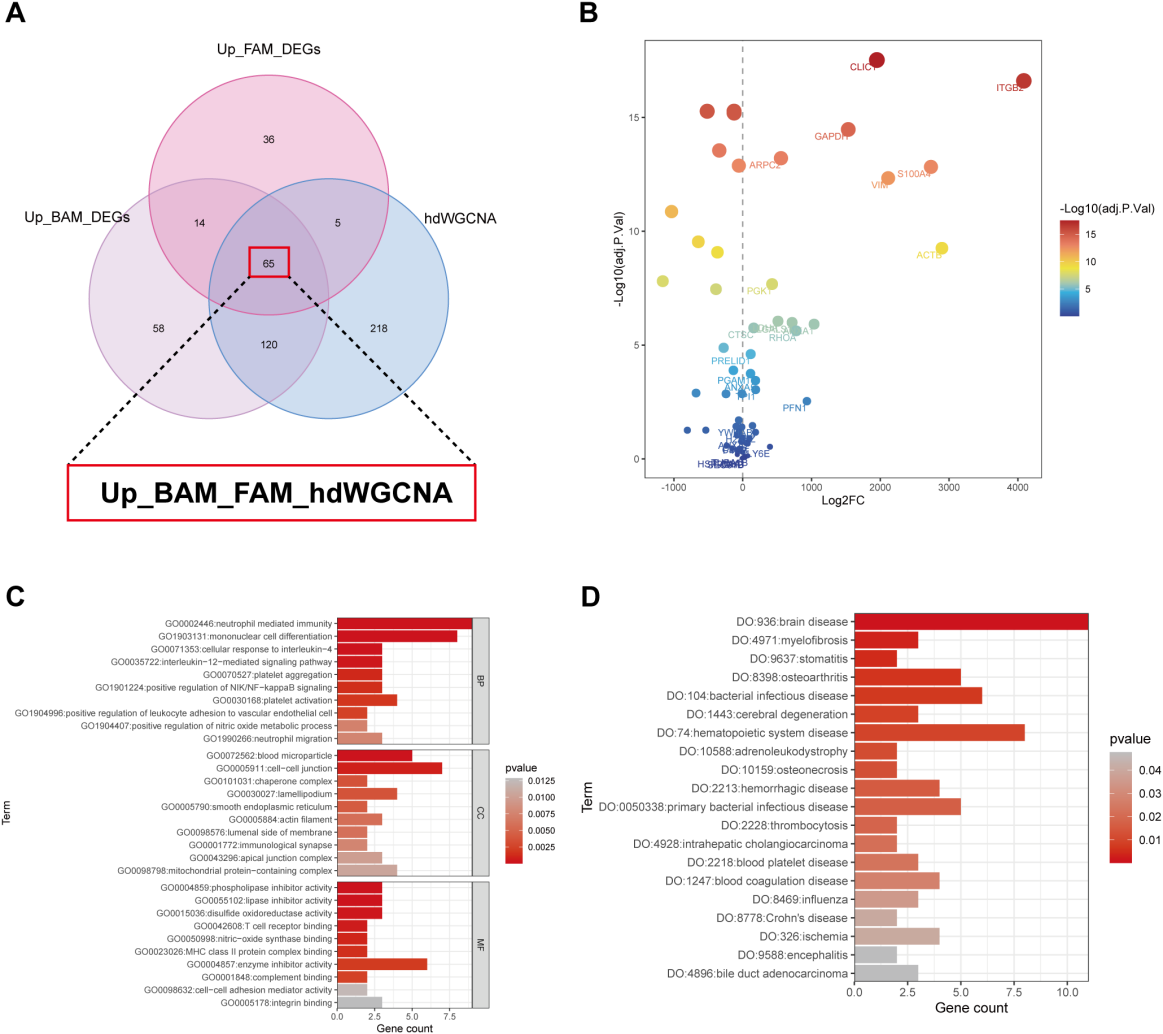
Characterization of the identified hub gene set. **(A)** The venn diagram obtained a hub gene set named Up_BAM_FAM_hdWGCNA which contained a total of 65 genes using differential expression gene (DEG) analysis and hdWGCNA. **(B)** Dot plot showing expression level of the 65 identified genes between KD patients and healthy controls. **(C)** Gene Ontology (GO) enrichment pathway of the hub gene set. **(D)** Disease Ontology (DO) enrichment pathway of the hub gene set. BP, biological process; CC, cellular component; MF, molecular function.

Through DEG analysis, we identified 257 BAM-related genes (**Figure 2E**) and 120 FAM-related genes (**Figure 2J**). Meanwhile, 408 high kME genes were obtained in the high-BAM and high-FAM cell group by hdWGCNA analysis (**Figure 3**). By taking the intersection in a Venn diagram, we discovered a hub gene set consisting of 65 genes named as Up_BAM_FAM_hdWGCNA, relating to the up-regulation of both BAM and FAM (**Figure 4A**, **Supplementary Table 8**). Among these 65 genes, only 61 genes demonstrated successful cross-platform matching in GSE68004 dataset, allowing for further analysis eventually. In these 61 genes, vimentin (VIM), integrin subunit beta 2 (ITGB2), chloride intracellular channel 1 (CLIC1), and S100A4 exhibited high expression levels (**Figure 4B**, **Supplementary Table 9** **).**

In order to elucidate the relationship between the genes of Up_BAM_FAM_hdWGCA and their roles in various biological processes and disease states, we reviewed the relevant literatures, which revealed that most genes were associated with BAM and FAM, as detailed in **Supplementary Table 8**, further supporting our findings. For further analysis, all 61 genes were imported into the STRING database. The query was restricted to “Homo sapiens,” and genes without known associations were excluded. The results of PPI analysis demonstrated that these hub genes were highly interconnected and exhibited strong functional correlations (**Supplementary Figure 3**). GO and DO analysis were performed to further confirm our results (**Figures 4C,D**, **Supplementary Tables 10**, **11**). The GO analysis indicated that, within the biological process (BP) category, these genes were upregulated in platelet aggregation and activation, neutrophil mediate immunity, and mononuclear cell differentiation. In terms of molecular function (MF), pathways related to phospholipase and lipase inhibitors activity, as well as integrin binding, also exhibited an upregulation trend. The DO analysis revealed upregulation in pathways associated with inflammatory-responsive diseases like Crohn’s disease and osteoarthritis, infectious diseases including bacterial infections disease and influenza, and thrombocytosis, which may share common pathogenic mechanisms with KD.

### 3.4 Machine learning algorithms identified signature genes

**Figure 5:**
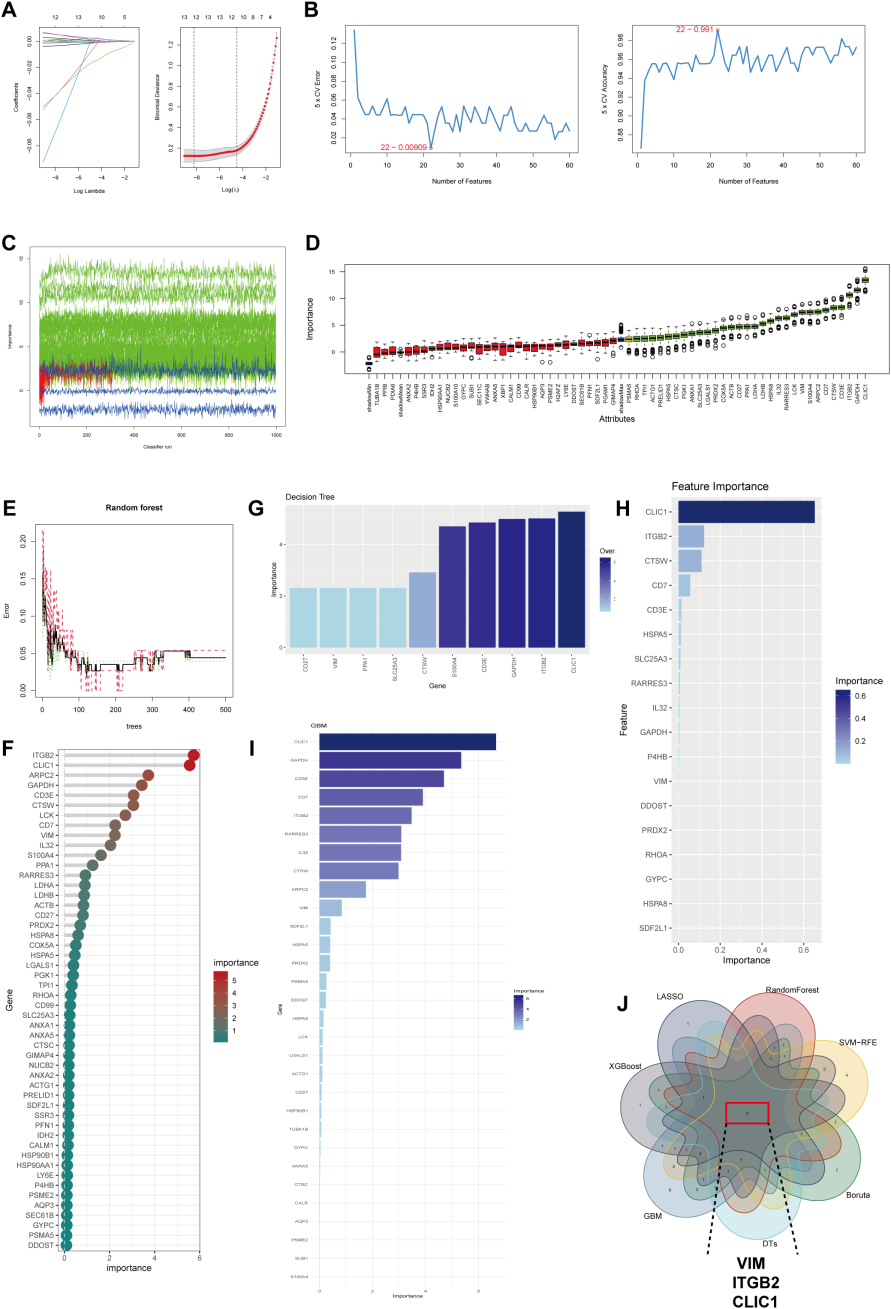
Machine learning identifies signature genes with high BAM and FAM in KD. **(A)** LASSO algorithm for selection genes related with elevated BAM and FAM. **(B)** The results of SVM-RFE algorithm for selection key genes. **(C, D)** The Boruta algorithm obtained 30 genes up-regulating BAM and FAM. **(E, F)** Results from the Random Forest (RF) algorithm. **(G)** The results of the Decision Tree (DT) algorithm. **(H)** The xGBoost algorithm selecting 18 key genes. **(I)** Gradient Boosting Machine (GBM) algorithm showing 30 genes were correlated to high BAM and FAM. **(J)** Venn diagram showing the signature genes between seven machine learning algorithms: VIM, ITGB2 and CLIC1.

Seven machine learning algorithms were employed to identify potential biomarkers for KD diagnosis. The LASSO algorithm identified 12 key genes (**Figure 5A**), whereas the SVM-RFE algorithm identified 22 key genes (**Figure 5B**). The Boruta algorithm selected 30 significant variables following 500 iterations (**Figures 5C, D**). Subsequently, the RF algorithm pinpointed 12 genes, each with an importance score >0 (**Figures 5E, F**). The decision tree (DT) algorithm identified 10 important genes (**Figure 5G**), while the xGBoost algorithm detected 5 key genes (**Figure 5H**). Furthermore, the GBM algorithm identified 30 key genes related to BAM and FAM upregulation (**Figure 5I**). The genes identified by these algorithms are listed in **Supplementary Table 12**. Ultimately, by integrating the gene sets from all seven machine learning algorithms, we acquired three optimal diagnostic genes: VIM, ITGB2 and CLIC1 (**Figure 5J**).

### 3.5 Biological function analysis of signature genes

In order to reveal distinct characteristics between the signature genes and their potential biological processes of interest, KEGG pathway enrichment analysis using GSEA was performed on the GSE68004 dataset. Two commonly enriched pathways were identified: primary bile acid biosynthesis and fatty acid metabolism KEGG pathways (**Supplementary Figures 4A–C**), consistent with previous studies (44–46). These findings indicate that BAM and FAM biological pathways were relevant to the pathogenesis of KD.

### 3.6 Expression landscape of the signature genes at single-cell level

**Figure 6:**
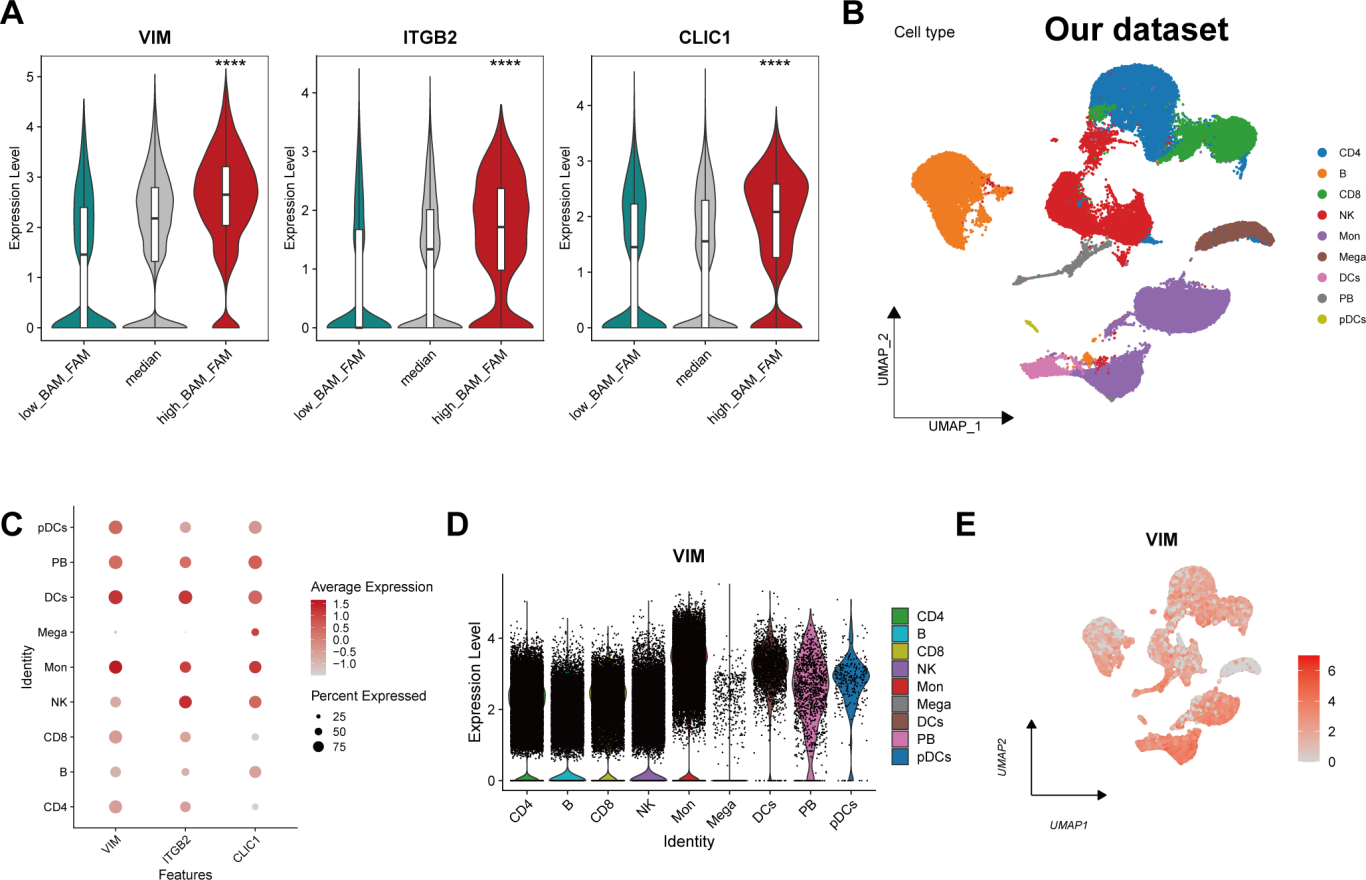
Expression landscape of the signature genes at single-cell level. **(A)** The violin plot showing the expression level of the characteristic genes in different metabolic cell groups. **(B)** Uniform manifold approximation and projection (UMAP) projections for cell clustering identified 9 cell types in our own scRNA-seq data. **(C)** Bubble plot showing the expression of VIM, ITGB2 and CLIC1 of each cell type. Each dot is colored by the expression and sized by the fraction of cells expressing the gene in the specific cell type. **(D)** Violin plot depicting the expression level of VIM in different cell subtype. **(E)** The UMAP plot showing the distribution of VIM across different cell types. ****p ≤ 0.0001.

VIM, ITGB2 and CLIC1 exhibited significantly increased expression in cells with simultaneous upregulation of both BAM and FAM (**Figure 6A**), implying their important role in regulating BAM and FAM. To validate these findings, scRNA-Seq analysis was conducted on our own dataset. After dimensionality reduction and clustering analysis, nine distinct cell clusters were identified based on classical marker genes (**Figure 6B**, **Supplementary Figures 5A–E**). The distribution of each cell type across different groups (HC, KD, KL) was shown in **Supplementary Figure 5F**, demonstrating an increase of monocytes in KD and KL. To further identify the specific cell types in which the characteristic gene function, we conducted a validation at the single-cell level. The results showed that VIM and CLIC1 were both highly expressed in monocytes (**Figures 6C–E**, **Supplementary Figures 6A, B**), whereas ITGB2 was predominantly expressed in NK cells (**Figure 6C**, **Supplementary Figures 6C, D**).

### 3.7 Trajectory analysis and cell interactions

**Figure 7:**
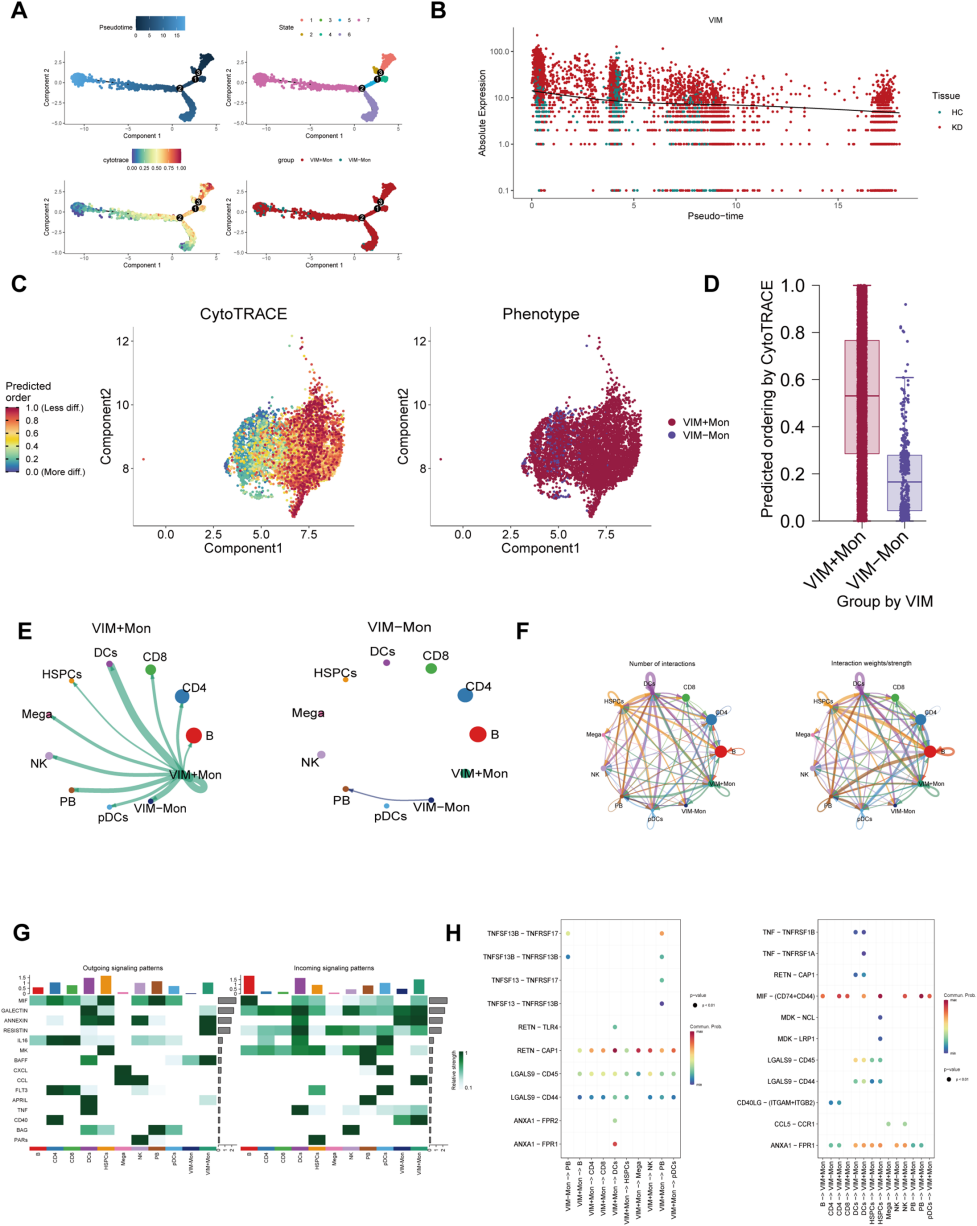
The landscape of cell trajectory and cell-cell communication of VIM. **(A)** Quasi-temporal analysis showing the change of proportions of VIM+ monocytes. **(B)** The results of pseudotemporal analysis portrayed the relative expression of VIM. **(C)** CytoTRACE analysis illustrated cell differentiation level in VIM labeled monocytes. **(D)** Bar graph showing the differentiation status of VIM labeled monocytes. **(E, F)** Circle plots showing the quantity and intensity of interactions between VIM^+^ monocytes and other cell types. **(G)** The heat map depicting the efferent or afferent contributions of all signals to different groups of cells. **(H)** The bubble chart shows ligand–receptor interactions. Bubble size represents *p* value generated by the permutation test, and the color represents the possibility of interactions.

Given the transcriptional heterogeneity of cells in KD patients, we employed pseudo-time analysis to examine monocytes marked by VIM and CLIC1, and as well as NK cells marked by ITGB2. Notably, the proportion of VIM^+^ and CLIC1^+^ monocytes, along with ITGB2^+^ NK cells, gradually increased over time (**Figure 7A**, **Supplementary Figures 7A**, **8A**). **Figure 7B** illustrated the relative expression of VIM in the pseudo-time analysis, while the results for CLIC1 and ITGB2 are presented in **Supplementary Figures 7B**, **8B**. These findings offer insights into the transcriptional heterogeneity of monocytes and NK cells in KD patients. Consistent with the pseudo-time analysis results, CytoTrace analysis revealed that VIM^+^, CLIC1^+^ monocytes and ITGB2^+^ NK cells exhibited a higher degree of differentiation status compared to VIM^-^, CLIC1^-^ monocytes and ITGB2^-^ NK cells (**Figures 7C, D**, **Supplementary Figures 7C, D**, **8C, D**).

To further investigate differences in intercellular communication between VIM^+^ and VIM^-^ monocytes, we analyzed the expression profiles of receptors and ligands. **Figures 7E, F** indicate that VIM^+^ monocytes exhibited more efficient signal transmission than VIM^-^ monocytes. Intercellular communication analyses involving CLIC1^+^ monocytes and ITGB2^+^ NK cells with other cell types highlighted the extent and intensity of these interactions (**Supplementary Figures 7E, F**, **8E, F**). **Figure 7G** illustrated the heightened communication capacity of VIM^+^ monocytes, characterized by distinct outgoing signaling patterns (e.g., ANNEXIN, RESISTIN, and BAFF) and incoming signaling patterns (e.g., GALECTIN, ANNEXIN, CCL, and CD40). Moreover, **Figure 7H** demonstrated the binding of VIM^+^ monocytes to different cell types via receptor-ligand pairs such as RETN−CAP1, MIF−(CD74+CD44) and ANXA1−FPR1. Similar findings were observed in CLIC1^+^ monocytes (**Supplementary Figures 7G, H**). Conversely, ITGB2+ NK cells exhibited outgoing signaling patterns involving MIF, ANNEXIN, IL16, CCL, and PARs, while their incoming signaling patterns included GALECTIN, RESISTIN, MK, and BAG (**Supplementary Figure 8G**). Notably, beyond their similarities with VIM+ monocytes, ITGB2+ NK cells also engaged in key ligand-receptor interactions like LGALS9−CD45 and MIF−(CD74+CXCR4) (**Supplementary Figures 8H**).

### 3.8 Validation of the signature genes and evaluation of their diagnostic efficacy

**Figure 8:**
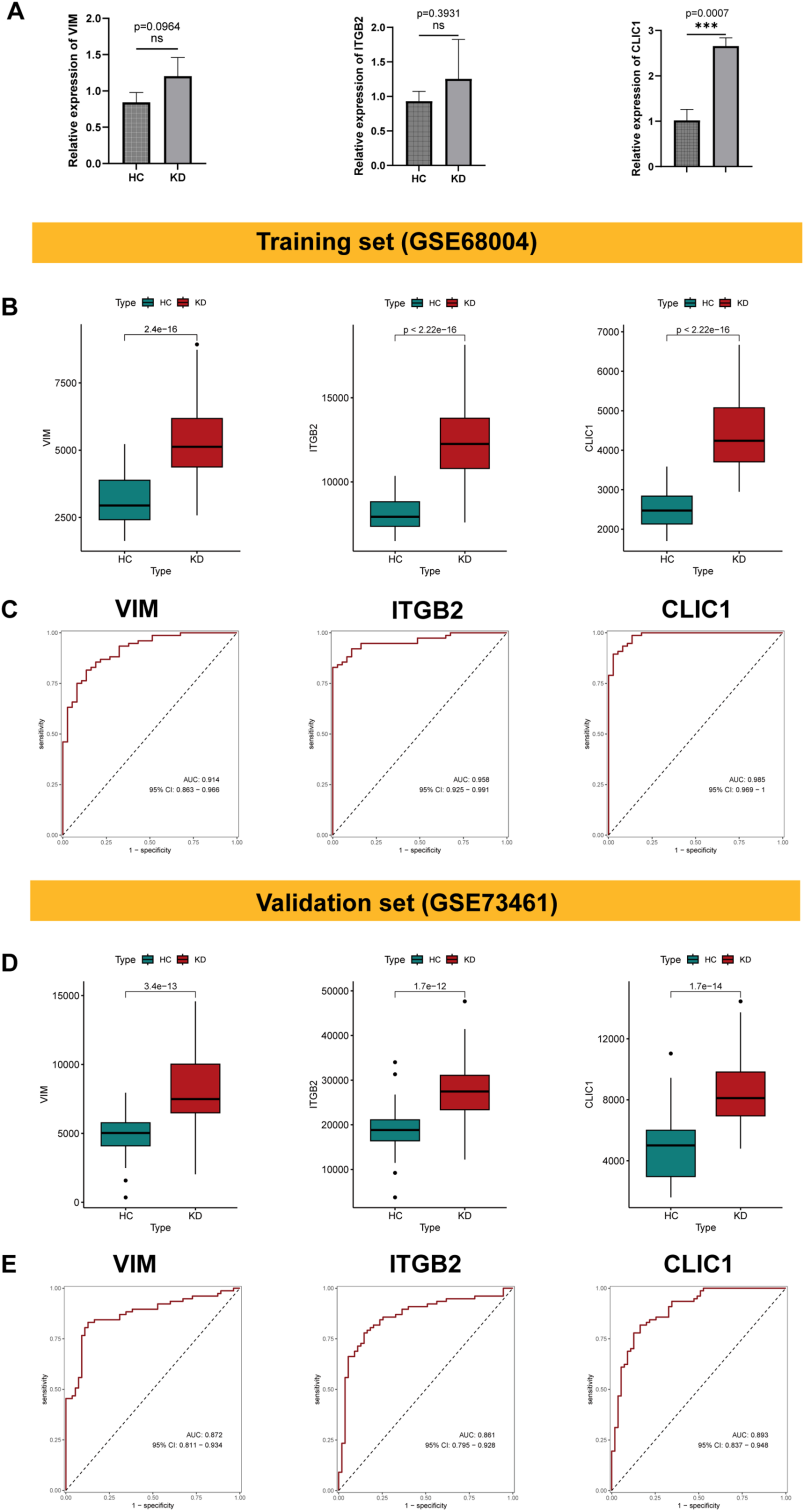
Validation of the signature genes and evaluation of their diagnostic efficacy. **(A)** The box plot showing the relative expression of VIM, ITGB2 and CLIC1 in peripheral blood mononuclear cells (PBMCs) from KD patients compared to healthy controls. Significant differences were determined with a two-sided Student’s T-test (***p<0.001, ns *p*>0.05). **(B)** Box plot showing the expression level of VIM, ITGB2, and CLIC1 in KD and healthy controls within the training dataset (GSE68004). **(C)** The ROC score and 95% confidence interval (CI) of the characteristic genes were present in the training dataset. **(D, E)** Validation of expression level of VIM, ITGB2 and CLIC1, along with corresponding ROC curves and 95% confidence interval (CI) in the validation dataset (GSE73461).

The qRT - PCR results showed that VIM, ITGB2 and CLIC1 were up-regulated in KD patients compared with healthy controls (**Figure 8A**), consistent with the bioinformatic analysis. To evaluate the expression level and diagnostic value of the signature genes, we initially performed analysis on the training set (GSE68004). VIM, ITGB2, and CLIC1 exhibited significantly higher expression in KD patients than healthy controls (*p* < 0.001, **Figure 8B**). Moreover, these genes demonstrated significant diagnostic potential. In the training dataset (GSE68004), the area under the curve (AUC) values and 95% CI were as follows: VIM: 0.914 (0.863–0.966), ITGB2: 0.958 (0.925–0.991), and CLIC1: 0.985 (0.969–1) (**Figure 8C**). The validation dataset (GSE73461) yielded similarly robust results, with AUC values and 95% CI: VIM: 0.872 (0.811–0.934), ITGB2: 0.861 (0.795–0.928), and CLIC1: 0.893 (0.837–0.948) (**Figures 8D,E**). These findings further validate our analysis and highlight the potential of these genes as diagnostic biomarkers for KD.

## 4 Discussion

KD, a self-limited multisystemic vasculitis, has been shown to result from activated immune and inflammatory responses, particularly within monocytes, in genetically susceptible children after exposure to the pathogenic agent or agents in the environment (47, 48), especially within monocytes. Dysregulation of BAM and FAM is closely associated with changes in immune status, as well as alterations in cell adhesion and migration, and other related functions (4, 49, 50). Studies have reported that bile acid and fatty acid levels were increased during the acute phase of KD (7, 51–53), and are associated with treatment response (6) and the development of CALs (7). However, a comprehensive understanding of BAM and FAM at the single-cell level in KD remains lacking. Here, by integrating machine learning algorithms and multi-omics data, our study for the first time found that the change of BAM and FAM in KD, suggesting the potential crosstalk between these metabolic pathways. Subsequently, three BAM-related and FAM-related signature genes: VIM, ITGB2 and CLIC1 were identified. These genes were found to be upregulated in cell groups with high BAM and FAM. Moreover, our own scRNA-seq data confirmed that VIM and CLIC1 were elevated in monocytes, while ITGB2 was upregulated in NK cells. qRT-PCR results further validated these bioinformatic findings. At the bulk-RNA level, these genes also demonstrated high diagnostic value for KD. Hence, our findings provided novel metabolism-related biomarkers for KD diagnosis and offer new insights into the metabolic influences on KD pathogenesis.

The protein encoding gene VIM has been widely studied for its role in regulating numerous fundamental cellular processes associated with various pathophysiological conditions, such as cataracts, Crohn’s disease, rheumatoid arthritis, HIV, and cancer. Its strong association with intracellular lipid metabolism (44) suggests a potential role in KD pathogenesis. Additionally, a previous study indicated that VIM regulates the activation of NLRP3 inflammasome, leading to increased IL-1β expression (54). Given the pivotal role of NLRP3 inflammasome in KD pathogenesis and vasculitis development (55, 56), VIM may contribute to the release of inflammatory mediators by modulating BAM and FAM.

ITGB2 (CD18), an adhesion molecule of the integrin family and a key marker of NK cells maturation, plays a crucial role in mediating adhesion between inflammatory cells and endothelial cells, inflammatory cell chemotaxis, and other processes involved in the early stages of atherosclerosis (57). Wang et al. demonstrated that the expression of ITGB2 was down-regulated following Tangzhiping intervention (45). Furthermore, immunofluorescence and ELISA experiments indicated a reduction in both adipose tissue and systemic inflammation in diabetic mice, supporting the hypothesis that ITGB2 may be involved in lipid metabolic inflammation. Additionally, ITGAM, which encodes the integrin alpha M chain in conjunction with ITGB2, exhibits elevated expression in acute KD (52). These findings provide a valuable foundation for further investigations into the role of ITGB2 in KD.

CLIC1, a member of the CLIC family, plays a crucial role in a number of important physiological functions, including cell cycle progression, differentiation and cell migration. Cholesterol-rich lipid rafts have been shown to enhance CLIC1 channel activity. Furthermore, the over-expression of CLIC1 has been demonstrated to raise the expression of several key proteins, including vascular endothelial growth factor, matrix metallopeptidase 2, matrix metallopeptidase 12 and matrix metallopeptidase 13 (46). Evidence from animal and cellular experiments indicate that CLIC1 contributes to the accelerated development of atherosclerotic plaques, increased oxidative stress, and the release of inflammatory cytokines *in vivo* (46). High-fat diet-fed mice showed CLIC1 overexpression in aortic tissues, while CLIC1-deficient human umbilical vein endothelial cells (HUVECs) displayed significantly reduced levels of tumor necrosis factor-α (TNF-α), interleukin-1β (IL-1β), intercellular cell adhesion molecule-1 (ICAM-1) and vascular cell adhesion protein 1 (VCAM-1) proteins (58). The similarities pathogenesis between KD and atherosclerosis suggests a potential role for CLIC1 in KD.

There are several limitations in our study. Firstly, there is population heterogeneity between the public scRNA-seq dataset and our in-house dataset. For instance, the public scRNA-seq dataset includes one case of incomplete KD, whereas all patients in our in-house dataset are complete KD. Additionally, our dataset includes four cases with CALs. The sample size and heterogeneity may introduce biases into our results. Secondly, in our qRT-PCR results, although VIM and ITGB2 showed an increasing trend, the differences were not statistically significant, possibly due to the small sample size of our PBMCs. Nevertheless, these findings offer preliminary insights for future research.

## 5 Conclusion

In conclusion, VIM and CLIC1 in monocytes, as well as ITGB2 in NK cells were identified as novel metabolism-related genes in KD. These signature genes might serve as biomarker for the diagnosis of KD.

## Data Availability

The datasets presented in this study can be found in online repositories. The names of the repository/repositories and accession number(s) can be found in the article/**Supplementary Material**.
