# Supplementary material for: Identification of novel metabolism-related biomarkers of Kawasaki disease by integrating single-cell RNA sequencing analysis and machine learning algorithms
Source: Front Immunol. 2025 Apr 10;16:1541939. doi: 10.3389/fimmu.2025.1541939 (PMC12018418; doi:10.3389/fimmu.2025.1541939)
Supplement: Supplementary file 2 [file DataSheet1.pdf]

Supplemental Materials for

Identification of novel metabolism-related biomarkers of Kawasaki disease by integrating single-cell RNA sequencing analysis and machine learning algorithms

Chenhui Feng[1], Zhimiao Wei[2], Xiaohui Li[1,2*]

*** Correspondence to:**

Xiaohui Li, MD, PhD.

Department of Cardiovascular Medicine, Children's Hospital Capital Institute of Pediatrics, No.2, Yabao Road, Chaoyang District, Beijing, China

Email: lxhmaggie@pumc.edu.cn

The file includes: Supplementary Figure 1-7 with their legends

Supplementary Figure 1-7 with their legends

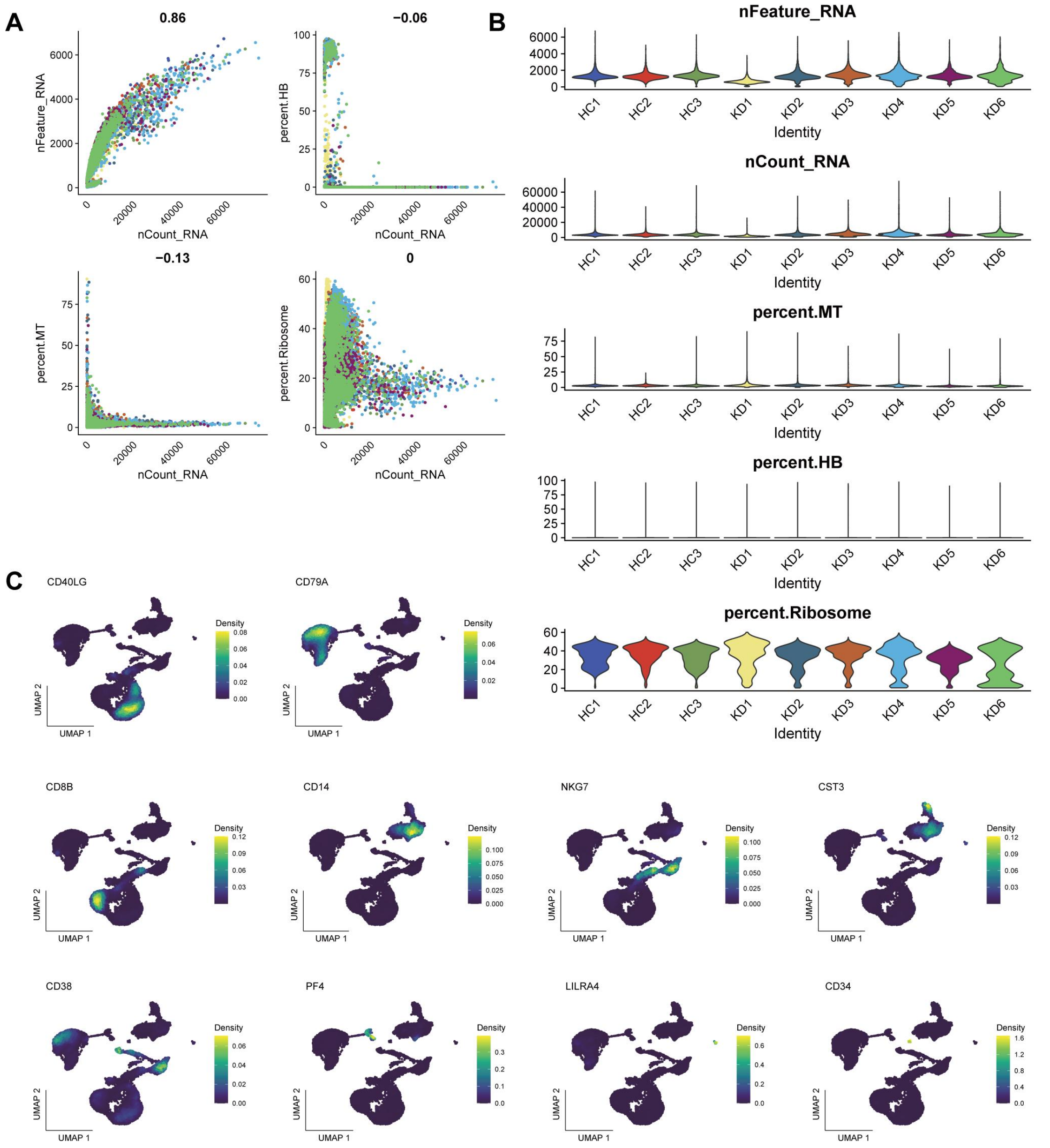

Supplementary figure 1. The fundamental characteristics of immune cells of single-cell RNA sequencing dataset GSE168732.

(A-B): The scatter plot and violin plot showing filtering and processing of the dataset GSE168732. (C) Uniform manifold approximation and projection (UMAP) showing typical marker genes for each cell group.

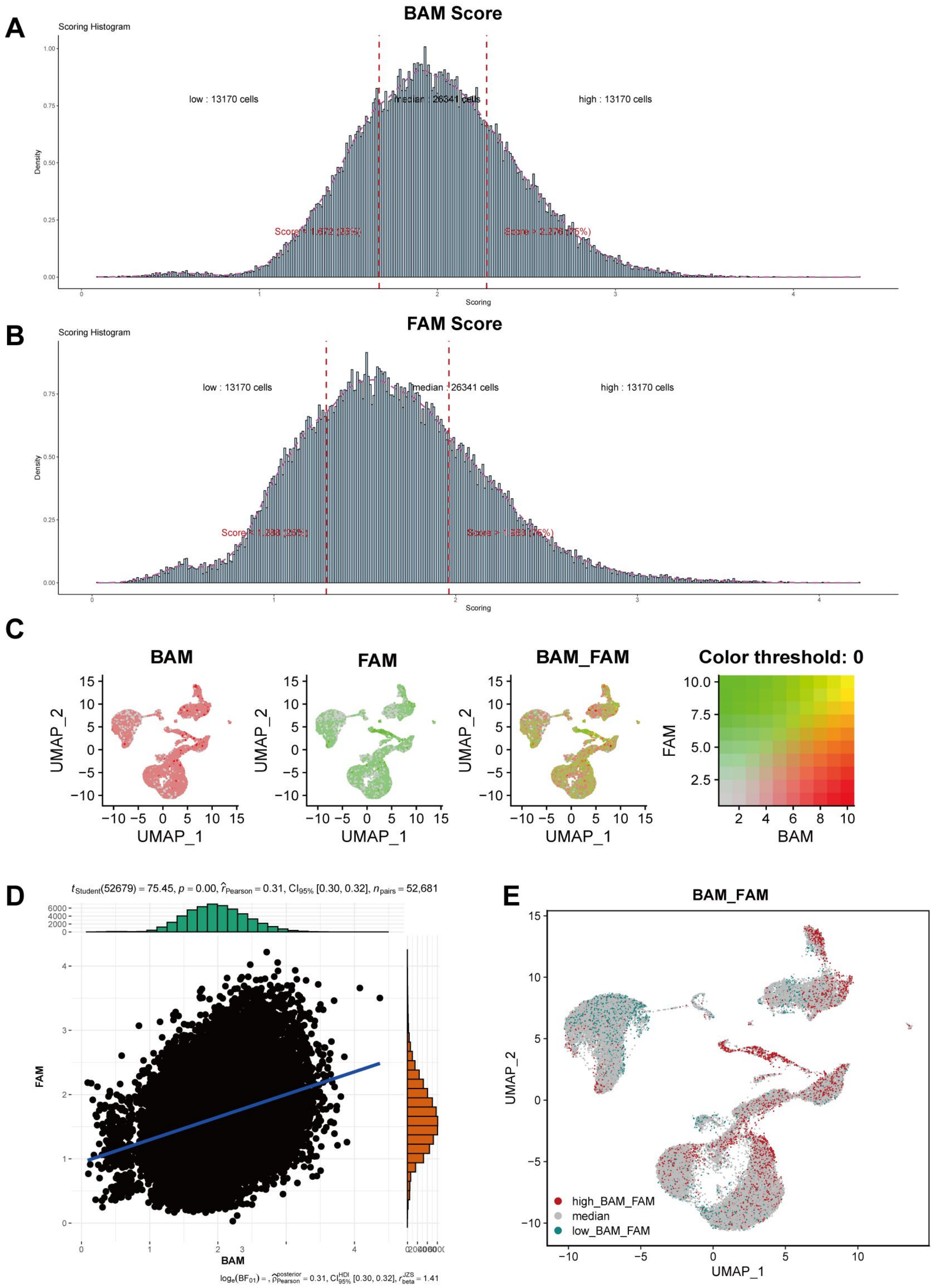

Supplementary figure 2. The metabolic scores of all cells calculated by quartile value and the correlation analysis between them.

(A) : The sinogram showing the BAM scores of all cells. (B): The sinogram showing the FAM scores of all cells. (C-D): The UMAP plot and scatter diagram showed the correlation between high BAM and FAM cells. **(**E**):** The UMAP plot showed three cells groups: high_BAM_FAM cells, low_BAM_FAM cells and median cell groups.

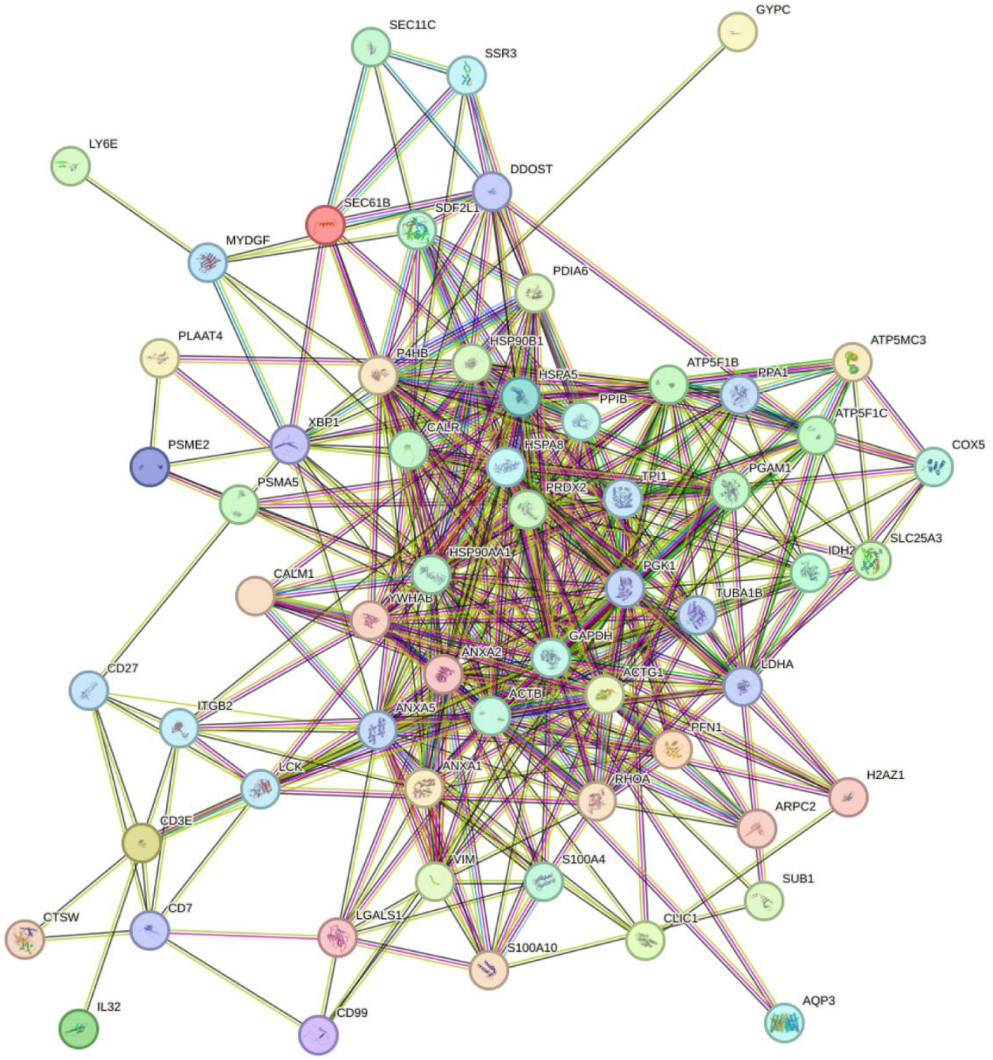

Supplementary figure 3. PPI network of the 61 hub genes.

The results of PPI analysis of the hub genes of BAM and FAM upregulation.

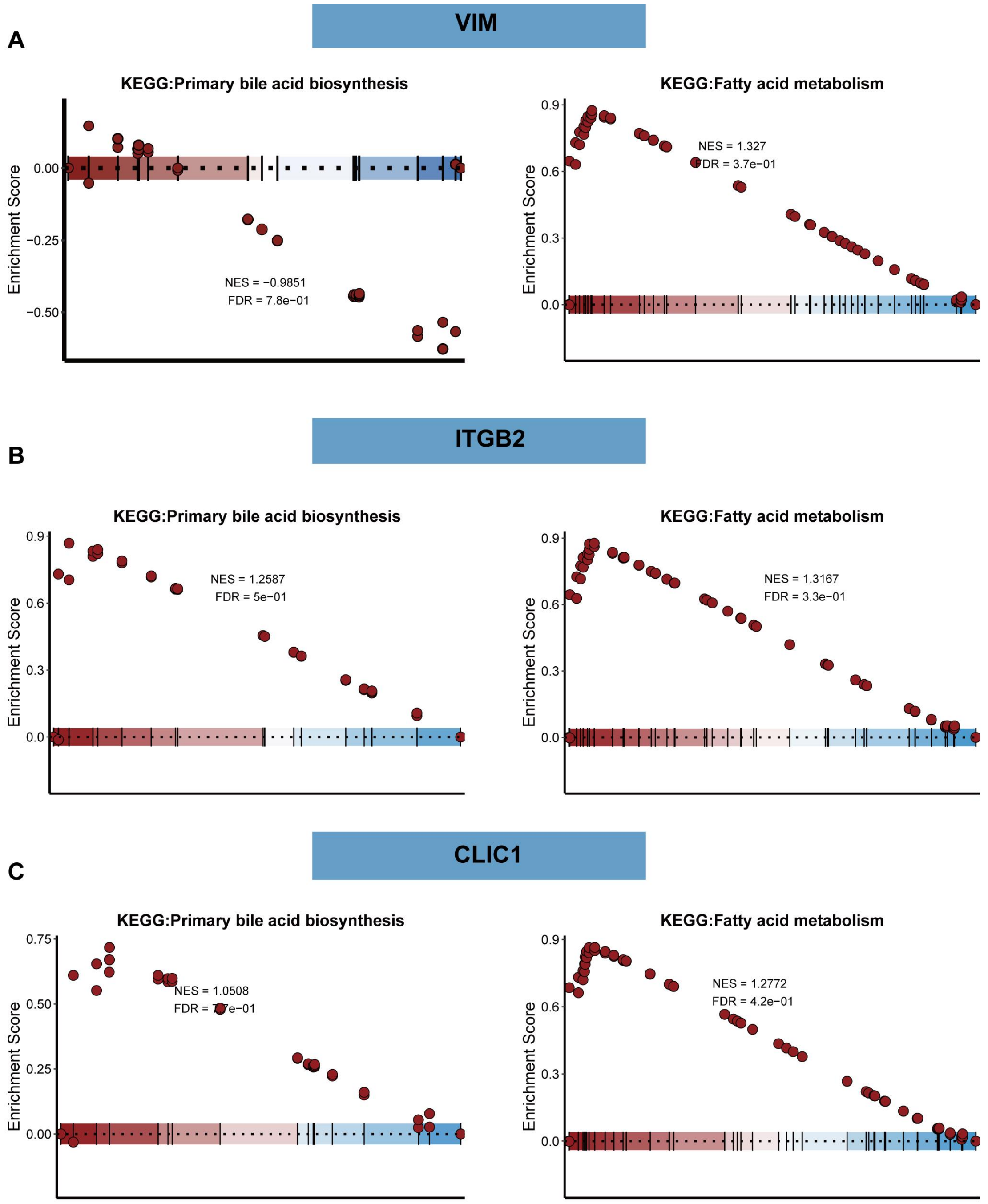

Supplementary figure 4. Single gene Gene Set Enrichment Analysis (GSEA) for the three signature genes.

(A) : Gene sets enrichment analysis (GSEA) identifies related KEGG pathway that are significantly enriched in the high expression of VIM. (B): GSEA showing related KEGG pathway that are significantly enriched in the high expression of ITGB2. (C): GSEA identifies related KEGG pathway that are significantly enriched in the high expression of CLIC1.

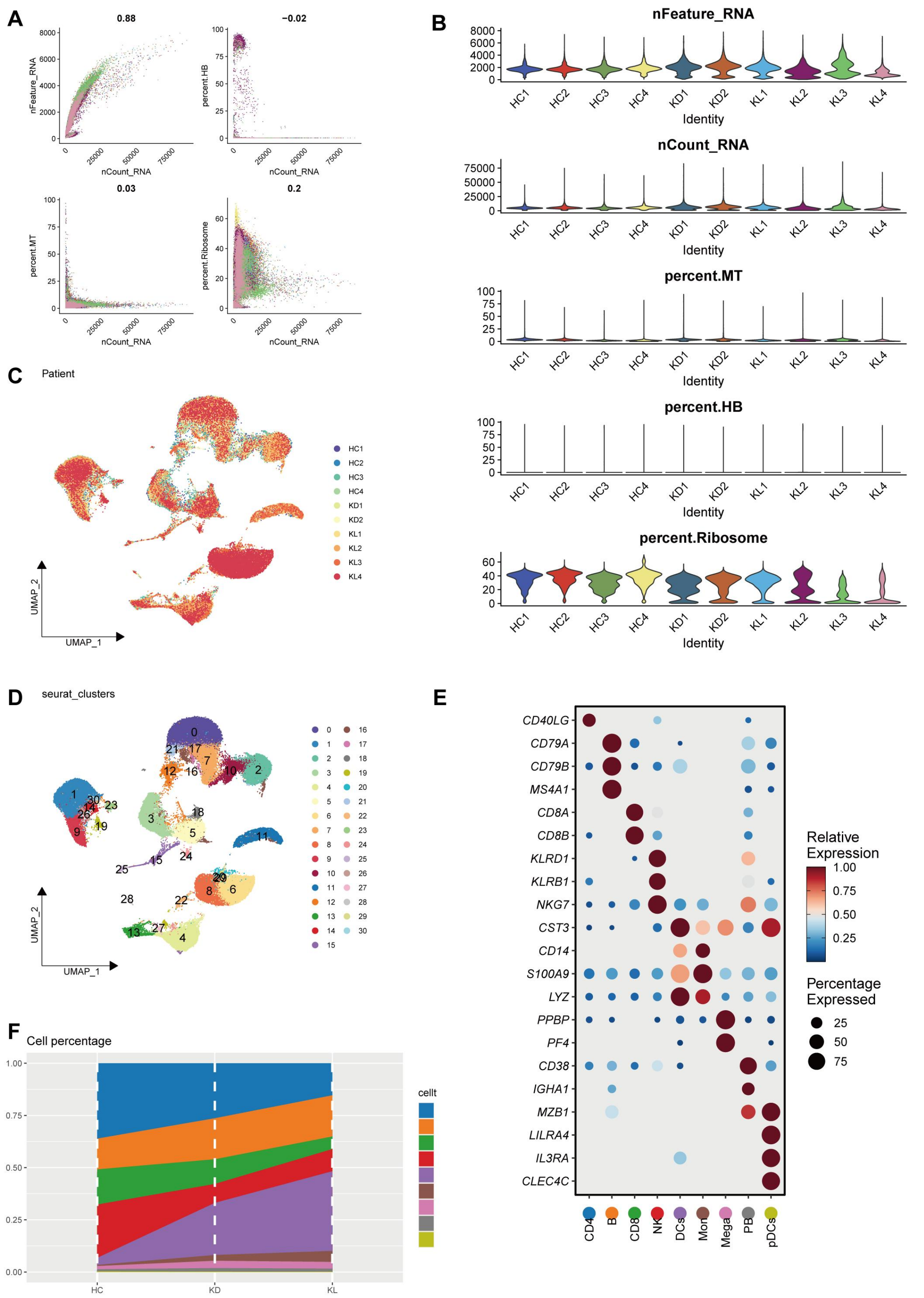

Supplementary figure 5. Signature of immune cells of our single-cell RNA database.

(A-B): The scatter plot and violin plot showing filtering and processing of the our dataset. (C): PCA demonstrated a consistent cell distribution across all analyzed samples. (D): Uniform manifold approximation and projection (UMAP) revealed a meticulous classification of 30 distinct clusters. (E): Dot plot showing typical marker

genes for each cell type. (F): The proportion of each cell type between KD, KL and healthy controls. HC, healthy control; KD, samples from patients with Kawasaki disease without coronary artery lesions; KL, patients with Kawasaki disease with coronary artery lesions.

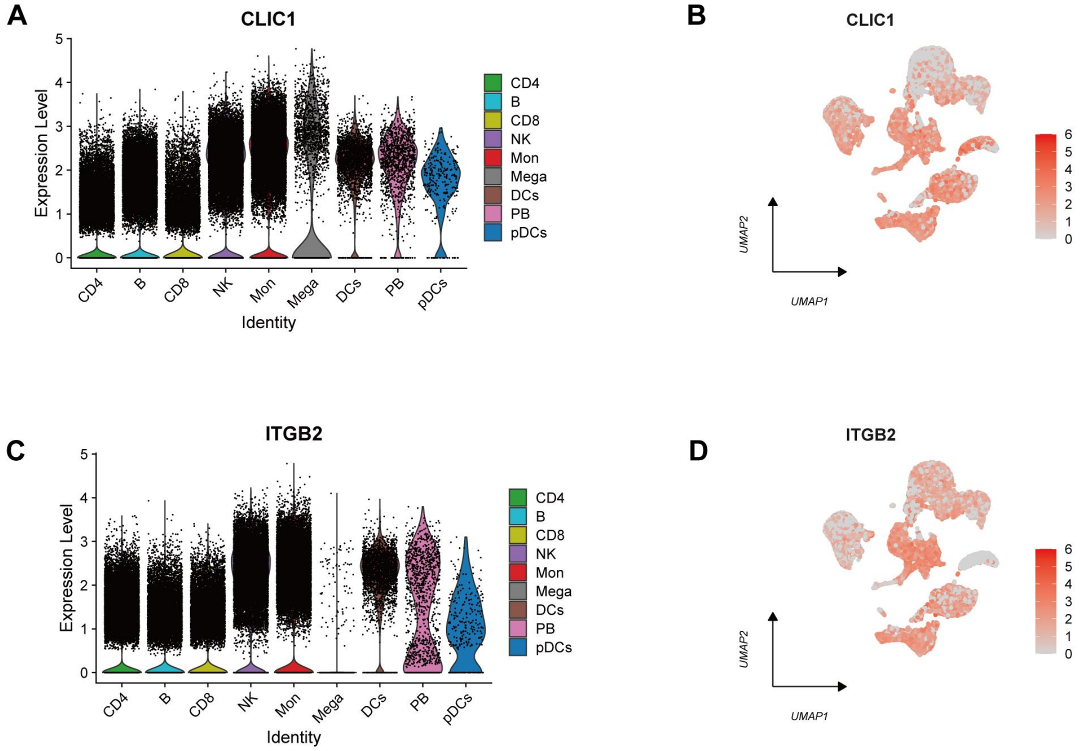

Supplementary figure 6. Characteristics of CLIC1 and ITGB2 expression and distribution.

(A) : Violin plot depicting the expression level of CLIC1 in different cell subtype. (B): The UMAP plot showing the distribution of CLIC1 across different cell types. (C): Violin plot depicting the expression level of ITGB2 in different cell subtype. (D): The UMAP plot showing the distribution of ITGB2 across different cell types.

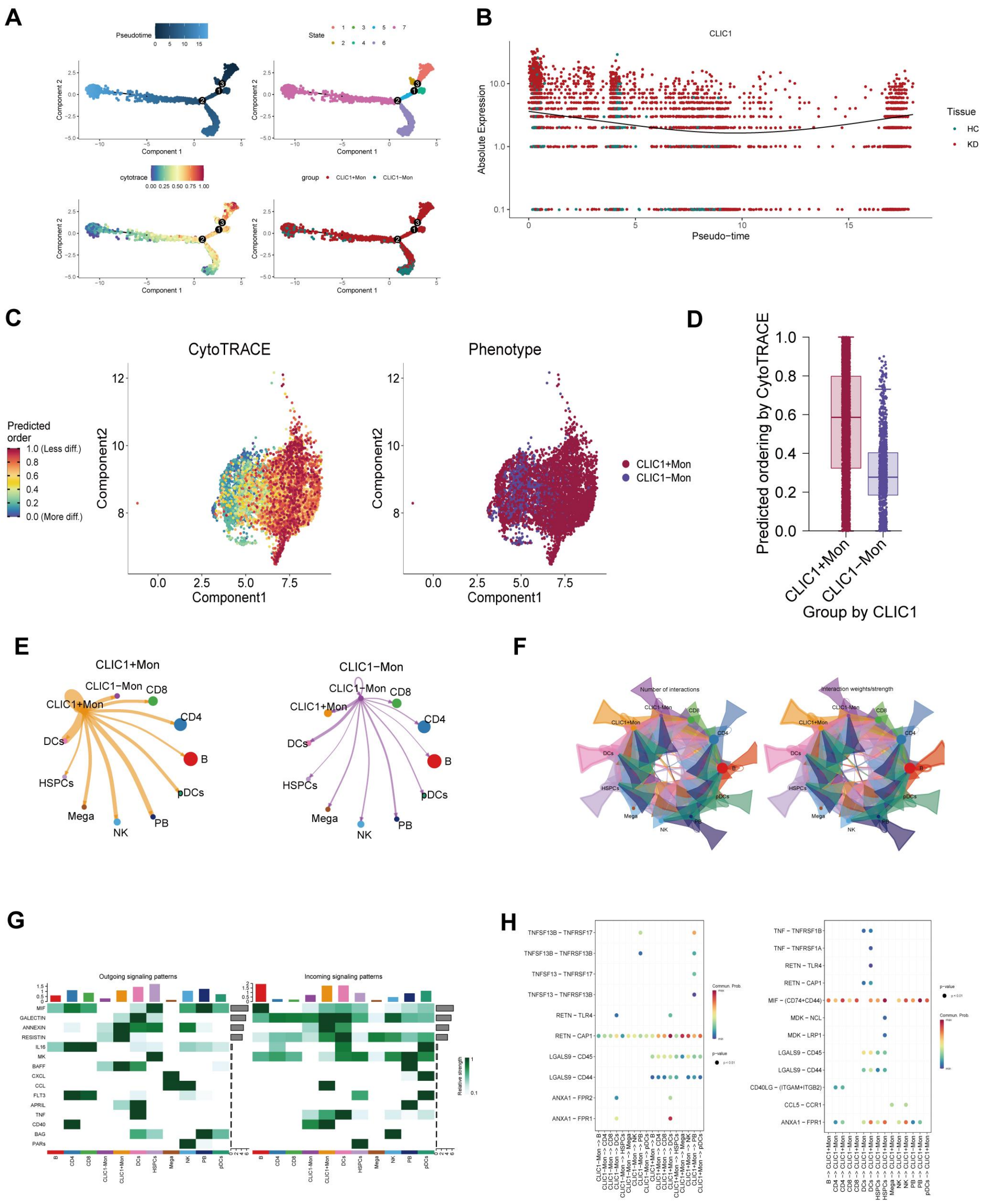

Supplementary figure 7. The landscape of cell trajectory and cell-cell communication of CLIC1.

(A) : Quasi-temporal analysis showing the change of proportions of CLIC1^{+} monocytes. (B): The results of pseudotemporal analysis portrayed the relative expression of CLIC1. (C): CytoTRACE analysis illustrated cell differentiation level in CLIC1 labeled monocytes. (D): Bar graph showing the differentiation status of CLIC1 labeled monocytes. (E-F): Circle plots showing the quantity and intensity of

interactions between CLIC1$^+$ monocytes and other cell types. (G): The heat map depicting the efferent or afferent contributions of all signals to different groups of cells. (H): The bubble chart shows ligand–receptor interactions. Bubble size represents *p* value generated by the permutation test, and the color represents the possibility of interactions.

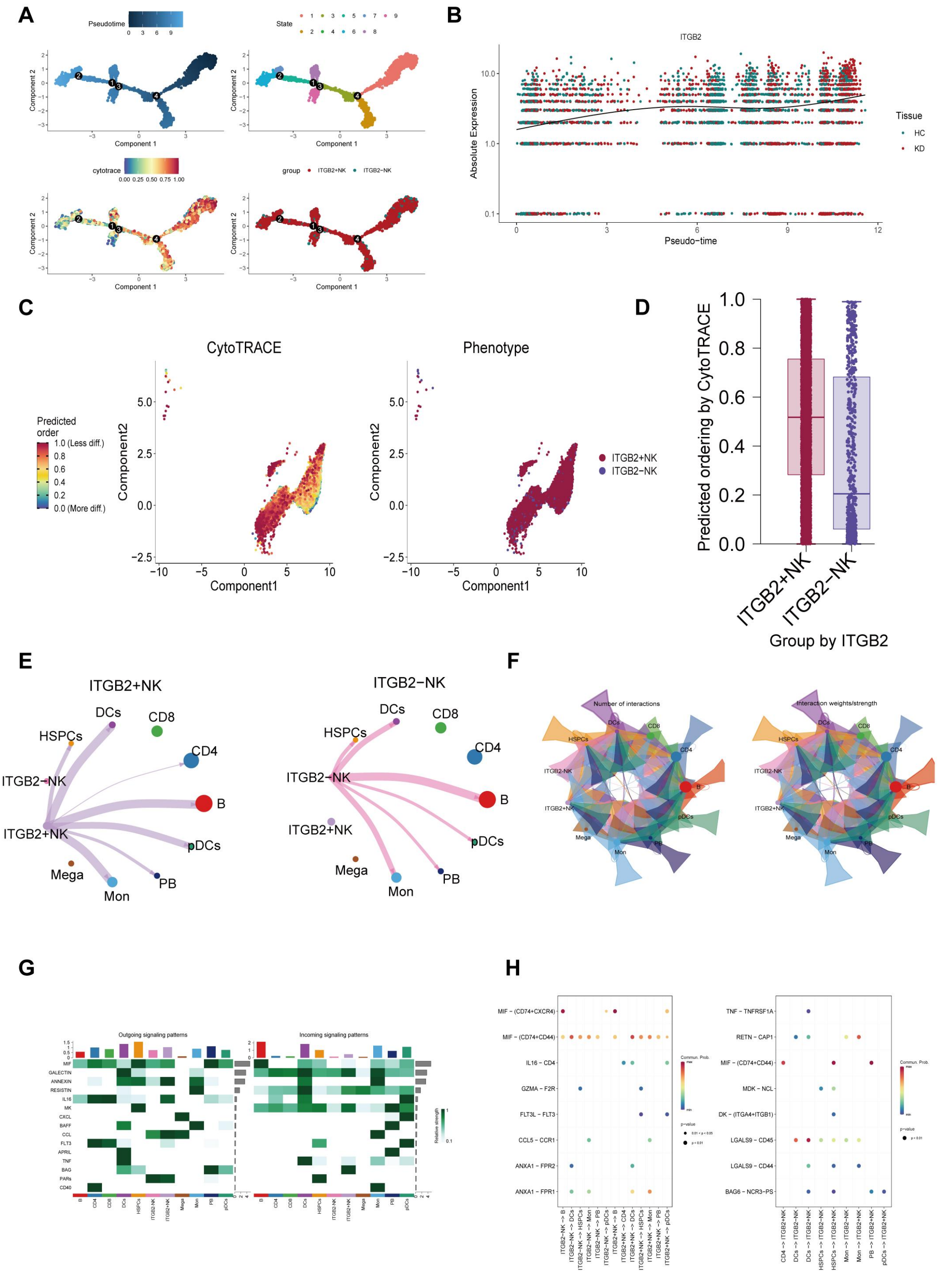

Supplementary figure 8. The landscape of cell trajectory and cell-cell communication of ITGB2.

(A): Quasi-temporal analysis showing the change of proportions of ITGB2^{+} NK cells. (B): The results of pseudotemporal analysis portrayed the relative expression of ITGB2. (C): CytoTRACE analysis illustrated cell differentiation level in ITGB2

labeled NK cells. (D): Bar graph showing the differentiation status of ITGB2 labeled NK cells. (E-F): Circle plots showing the quantity and intensity of interactions between $ITGB2^{+}$ NK cells and other cell types. (G): The heat map depicting the efferent or afferent contributions of all signals to different groups of cells. (H): The bubble chart shows ligand–receptor interactions. Bubble size represents *p* value generated by the permutation test, and the color represents the possibility of interactions.
